# Age-Related Change in the Daily Stressor Reactivity Across 20 Years of Adulthood

**DOI:** 10.1093/geroni/igab046.823

**Published:** 2021-12-17

**Authors:** David Almeida, Jacqueline Mogle, Jonathan Rush

**Affiliations:** 1 Pennsylvania State University, University Park, Pennsylvania, United States; 2 Penn State University, University Park, Pennsylvania, United States; 3 University of Victoria, Victoria, British Columbia, Canada

## Abstract

Affective reactivity to everyday stressful events has been shown to be an important predictor of poor mental and physical health. The purpose of this study was to examine longitudinal changes in daily stress across 30 years of adulthood as a critical first step for understanding aging-related trends in daily stress. We used data from the National Study of Daily Experiences (NSDE) to calculate exposure and reactivity to daily stressors collected during telephone interviews over the course of 8 consecutive days. These daily assessment bursts were conducted in 1997, 2007, and 2018. Data were comprised of 33,931 daily interviews from 2,880 adults ages 25-74 at the first burst. Results indicated decreased stressor reactivity over time but this decrease was greater for younger adults. Discussion will focus on how examining change in daily stress processes is critical for illuminating stress and health.

